# Influence of weight status on bone mineral content measured by DXA in children

**DOI:** 10.1186/s12887-021-02665-5

**Published:** 2021-04-20

**Authors:** Francisco Sánchez Ferrer, Ernesto Cortes Castell, Francisco Carratalá Marco, Mercedes Juste Ruiz, José Antonio Quesada Rico, Ana Pilar Nso Roca

**Affiliations:** 1 Medical School, University of Miguel Hernández de Elche, Sant Joan d’Alacant, Spain; 2 Hospital Universitario de San Juan de Alicante, Ctra N-332, s/n, 03550, Sant Joan d’Alacant, Alicante Spain

**Keywords:** Obesity, Bone mineral content, Bone mineral density, Densitometry, Weight status, Osteoporosis

## Abstract

**Introduction:**

Childhood obesity is a public health problem with repercussions in later life. As tissue formation peaks in childhood we determined how weight status influences bone mineral content.

**Material and methods:**

We studied 553 children aged 4–18 years over 10 years (46.8% girls). We measured age, weight, height and through bone densitometry (DXA), bone mineral content (BMC), bone mineral density (BMD), and waist, arm and hip circumferences. The patients were divided into groups using the body mass index z-score: underweight, normal weight, overweight, obese and very obese.

**Results:**

BMC and BMD values were highest in the normal-weight and overweight groups. Logistic regression showed bone mineralization was inversely associated with waist circumference, the association being positive for weight and age. No differences were found according to sex.

**Discussion:**

Studies of the relationship between weight and bone mineralization report contradictory results, often because of different study designs. Moreover, studies in children are either few or with small samples. Our findings in a large sample show the importance of weight status in bone mineralization given the risk of bone fractures or osteoporosis.

**Conclusions:**

Weight status influenced bone mineralization. BMC and BMD decreased in children with a higher degree of obesity. Waist circumference correlated negatively with bone mineralization.

## Introduction

In the past 3 decades, the prevalence of childhood obesity has more than doubled in children and tripled in adolescents; indeed, it is so serious that it is now considered a public health problem [1]. In the United States, the prevalence of obesity in adults is 39.8% [2]. In Spain, two thirds of adults and one third of children are overweight [3, 4]. Obesity is considered a chronic progressive disease with a high probability of generating co-morbidities and an impact on health. When obesity begins in childhood, it tends to persist in adolescence and later in life [5–7].

As bone tissue formation is at its peak in childhood, it is of great importance to understand the relationships. In adults, obesity appears to have a protective effect against osteoporosis, due to increased bone mineral density (BMD) and a decreased risk of certain fractures due to frailty but an increased risk of other types of fracture, which has been called the “obesity paradox.” [8, 9]. An extensive review of relevant articles shows that the effect of overweight and obesity in children is associated with an increased BMD, though its impact in adults is less clear [10]. Classical studies in children have demonstrated that total bone area increases mainly with height and weight [11, 12]. The question is whether the increase in (BMD) is sufficient to compensate for the greater mechanical load [7, 8, 13]. Studies in children are contradictory. According to some authors, adolescents with obesity or overweight have a higher bone mineral content (BMC) and BMD. In contrast, other studies report these bone mineralization rates as equivalent or lower when compared to normal-weight adolescents or those with a lower body fat percentage [13–15]. The problem with many of these studies is that they were carried out with a small sample and in a few, the BMC was adjusted in relation to the body mass index (BMI). The largest study by Duran et al. showed differences in the effects of age, gender, body fat and body height [16].

Recently, reference tables have become available for children and adolescents to undertake a correct densitometry assessment according to sex and race, thus helping interpret the data obtained in usual clinical practice [17].

Obesity in children and adolescents appears to be associated with an increased risk of bone fractures [18], which may be due to several factors in addition to vitamin D levels. One factor is body weight, which is strongly associated with the BMD of weight-bearing bones and, to a lesser extent, to non-weight-bearing bones. Another is sex, as the association between fat mass and BMD in girls is stronger than in boys. In addition, this noted sex difference could be caused by the production of estrogen through aromatization in adipose tissue [19]. Estrogen plays a more important role than androgen in bone maturation. Indeed, estrogen therapy in men with aromatase deficiency results in an increase in BMD [20].

Bone mineralization during the pediatric stages is of great importance, as bone growth in childhood and adolescence accounts for approximately half of the bone mass achieved in adulthood. Bone mineralization increases with age, height and body mass during childhood, peaking during puberty [13], and its deficit may contribute to an increased risk of osteoporotic fractures in the future [18, 21, 22]. Many factors, such as heredity, vitamin D status, and other lifestyle factors like diet, physical activity, and body composition, significantly influence bone development in childhood and adolescence [21].

Different techniques have been used to measure BMC and BMD [23], with dual energy x-ray absorptiometry (DXA) used to classify BMC and BMD in accordance with the criteria established by the WHO, and the incorporation of BMD into the WHO fracture risk assessment algorithm (FRAX) [24]. DXA is currently the most widely used technique to assess BMC and BMD in children in a highly accurate, quick and effective manner with moderate radiation exposure [7, 9, 19, 20] Although it is considered the gold standard for measuring body composition and for the diagnosis of osteoporosis [25], assessment by DXA may overestimate true BMD in larger than normal individuals and underestimate BMD in smaller than normal individuals [26]. Other measures such as bioelectrical impedance or air displacement plethysmography are not exactly interchangeable [27, 28].

Clearly, the study of the possible effect of overweight and obesity on BMD during the skeletal growth period is of great interest and, as we have seen, a great deal of controversy exists [29]. The aim of this study was therefore to determine the effect of weight status on bone mineral content measured by DXA in children.

## Material and methods

### Study population

This cross sectional, retrospective study included a convenience sample comprising 553 children and adolescents (aged 4–18 years of age), who visited the Nutrition, Growth and Metabolism Clinic at San Juan de Alicante University Hospital for nutrition-related problems over the last 10 years (between January 2010 and January 2020) and who had undergone DXA. The pediatric service of this hospital is a reference center for nutritional disorders in children in the health area, covering 34,626 inhabitants under 18 years of age.

### Variables and measures

The main variables studied were the BMC, BMD and the bone mineral percentage. The values for BMC and BMD were obtained using DXA, performed using a General Electric Lunar DXA densitometer model DPXN PRO™. This equipment uses pencil-beam technology with a fixed 76 KV source, enabling high-speed examinations, optimizing scans and providing accurate results. The same software was used throughout the study. All the measurements were made with the same device by the same technician. The device was calibrated daily prior to starting the sessions and received yearly technical revisions according to the provider’s instructions.

From the BMC and the weight of the patient, the bone mineral percentage was calculated: $\% Bone\ mineral=\frac{BMC\ (g)}{Weight\ (g)}x\ 100$.

As secondary variables, sex, age, height, weight and waist and hip circumferences of the patient in underwear and without shoes were analyzed with a stadiometer (measurement error 0.5 cm), a tape measure (measurement error 0.2 cm) and a Tanita® scale (measurement error 0.1 kg). All the measurements were made with the subject standing, using a non-deformable plastic tape. Measurements were made for the waist at the mid-point between the last rib and the iliac crest and for the hip in the mid-zone of the buttocks.

BMI values and BMI z-scores were calculated with the Seinaptracker program (Medicalsoft Intercath, S.L., University of Barcelona, 2007–2008) using as a reference the growth curves and tables by Ferrández et al. [30] To define children with overweight or obesity, the BMI z-score values of + 1.0 overweight (85th percentile), + 2.0 obese (97th percentile) and + 3.0 very obese (99th percentile) were applied as cut-off points. We also included the concept of underweight defined as a BMI z-score value of − 2 (3rd percentile) [31].

### Statistical analysis

Statistical analysis of the data was performed using IBM SPSS Statistics version 26.0, calculating measures of central tendency (mtextean) and dispersion (standard deviation) for the quantitative variables and absolute frequency and relative frequency for the qualitative variables. Student’s *t*-test was used to compare means and standard deviations between sexes. The Kruskal-Wallis test was used to compare weight groups (BMI z-score) and Pearson’s chi-square test was used to analyze the weight status distribution according to BMI z-score and sex. Finally, linear regression analysis was performed, sequentially eliminating those variables that could act as confounding factors (*p* > 0.2), obtaining the ANOVA correlation coefficients. The significance level used was *p* < 0.05.

With 553 subjects, following the rule of 15 subjects per parameter to be estimated, it is possible to adjust a multivariate linear model of 36 dichotomous quantitative or categorical explanatory variables. For a multivariate model of 5 explanatory variables, to detect an effect size of 0.85 (adjusted R2), with a significance of 0.05, the statistical power reached is 100%.

### Ethical issues

This study was conducted under the ethical principles of the Declaration of Helsinki. No tests or analyses not specified in standard clinical practice were performed on the patients. The anonymization and confidentiality of the data, which were used exclusively for statistical purposes, were assured at all times. The protocol was approved by the Clinical Research Ethics Committee of San Juan de Alicante University Hospital (ref. 16/305).

## Results

The study sample comprised 553 children and adolescents, of whom 53.2% were boys and 46.8% were girls. The mean age was 11.5 ± 2.8 years, with a minimum and maximum age of 4 and 18 years, respectively. The mean BMI z-score was 2.9 ± 1.6 for the boys and 3.0 ± 1.8 for the girls, clearly a sample of patients with obesity. The parameters analyzed are shown in Table 1. Bone mineral values measured in their different forms (BMC, BMD and bone mineral percentage by sex) are also shown in Table 1, with no differences between sex.

**Table 1:** Values of the variables analyzed by sex

Variable	Boys 294 (53.2%) mean ± SD; n (%)	Girls 459 (46.8%) mean ± SD; n (%)	***P*** value
Age (years)	11.68 ± 2.75	11.20 ± 2.74	0.044
Height (cm)	153.3 ± 15.9	148.8 ± 13.9	< 0.001
Weight (kg)	64.1 ± 19.0	59.5 ± 17.8	0.003
Waist (cm)	85.0 ± 11.4	79.8 ± 9.9	< 0.001
Hip (cm)	94.1 ± 12.0	92.6 ± 15.4	0.222
BMI z-score	2.96 ± 1.66	2.93 ± 1.79	0.819
Underweight	6 (2.0)	5 (1.9)	0.262
Normal weight	16 (5.4)	26 (10.0)	
Overweight	46 (15.6)	31 (12.0)	
Obese	97 (33.0)	87 (33.6)	
Very obese	129 (43.9)	110 (42.5)	
BMC (g)	2008.2 ± 637.9	1927.1 ± 642.7	0.141
BMD (g/cm^2^)	1.03 ± 0.12	1.02 ± 0.13	0.294
Bone mineral (%)	3.15 ± 0.49	3.22 ± 0.52	0.073

*BMC/BMD* total body scans to evaluate the bone density and content

When distributing the sample according to the BMI z-scores of the boys and girls, no significant differences were found between the weight status groups according to BMI z-score and sex (Pearson’s chi square = 5.254; *p* = 0.262).

When analyzing the values of the three variables that indicate bone mineralization (BMC, BMD and bone mineral percentage), the distributions by groups classified according to weight status are shown in the following figures.

**Fig. 1:**
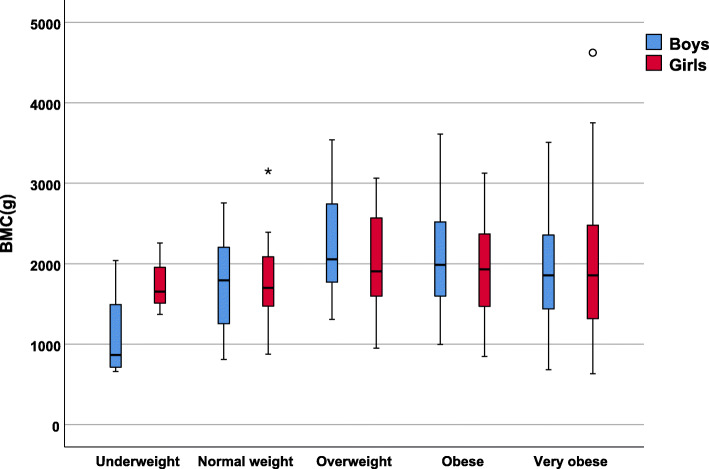
Total bone mineral content (BMC) values according to the weight status and sex of the study population

Figure 1 illustrates a lower BMC in children and adolescents classified as underweight. BMC is higher with weight status, rising higher in the children with normal weight and overweight, then lower in the children with obesity and extreme obesity. The comparison by sex (Fig. 1) shows that in the girls, BMC values were somewhat lower except in the underweight and very obese groups, where they had a higher BMC than the boys. However, these differences were not significant.

To determine whether the higher and subsequent lower BMC presented significant differences between the groups in relation to their weight status, the Kruskal-Wallis non-parametric test was performed, detecting significant differences (*p* = 0.002).

**Fig. 2:**
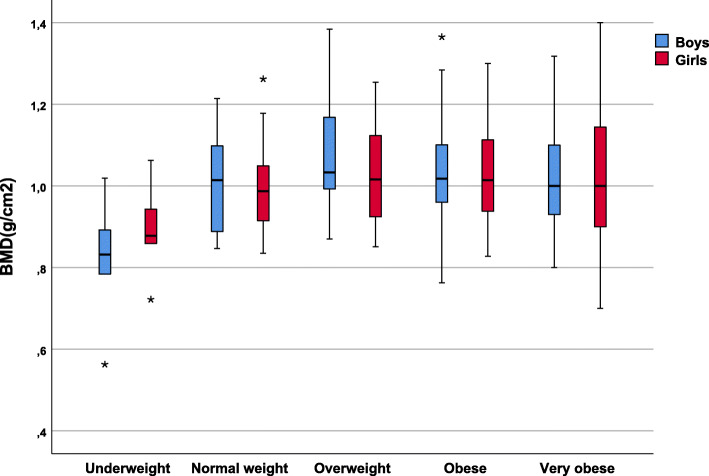
Bone mineral density (BMD) values according to weight status and sex

Figure 2 shows that the same occurred with the BMD values as with the BMC values. The BMD values are higher until the overweight state and decreased for the obese and very obese groups.

The non-parametric Kruskal-Wallis test revealed a difference in BMD values in the different weight status groups (*p* < 0.001), indicating that the increase in BMD for the normal weight and overweight groups, and subsequent decrease for the obese and very obese groups, was highly significant.

The same occurred with the BMD values according to sex; that is, they increased up to the overweight group and decreased in the obese and very obese groups. This trend was the same for boys and girls, with no significant differences by sex.

When analyzed in more detail, the BMD values in the girls were slightly lower than in the boys for the normal weight, overweight and obese groups. In the underweight group, girls had a higher BMD and in the obese group they had a similar BMD, but these differences were not significant.

**Fig. 3:**
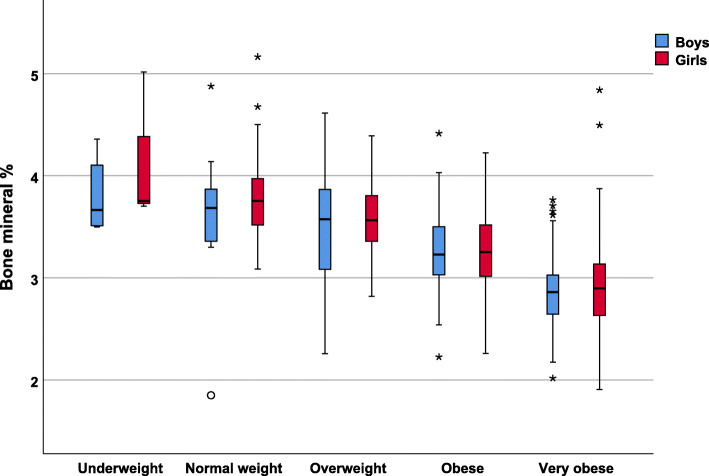
Bone mineral percentage values according to weight status and sex

The bone mineral percentage study (Fig. 3) indicated a decrease according to weight status measured by the significant BMI z-score, using the non-parametric Kruskal-Wallis test, which yielded a *p*-value < 0.001, with no significant differences by sex.

Logistic regression revealed that BMC increased with age, height, weight and was inversely proportional to waist circumference, with BMC also being higher in boys. In the case of BMD, we saw a positive correlation with age and weight and an inverse correlation with waist circumference (Table 2).

**Table 2:** Linear regression analysis of both the total sample (*n* = 553) and by sex (boys *n* = 294; girls *n* = 259) of the variables that showed significance with bone mineral content values and bone mineral density values

	Bone mineral content β (95% CI; *p* value β)			Bone mineral density β (95% CI; *p* value β)		
	Total	Boys	Girls	Total	Boys	Girls
Adjusted *R*^2^ (p Anova)	0.852 (< 0.001)	0.852 (< 0.001)	0.859 (< 0.001)	0.712 (< 0.001)	0.667 (< 0.001)	0.760 (< 0.001)
Age (years)	34.5 (18.4–50.6; < 0.001)	19.9 (−2.7–42.5; < 0.001)	44.2 (21.5–66.9; < 0.001)	0.016 (0.012–0.019; < 0.001)	0.016 (0.011–0.021; < 0.001)	0.016 (0.012–0.020; < 0.001)
Height (cm)	17.8 (13.9–21.6; < 0.001)	24.2 (18.8–29.5; < 0.001)	13.5 (7.9–19.0; < 0.001)	NA	NA	NA
Weight (kg)	20.2 (16.6–23.9; < 0.001)	14.7 (9.8–19.5; < 0.001)	24.1 (18.4–99.7; < 0.001)	0.005 (0.004–0.006; < 0.001)	0.005 (0.003–0.006; < 0.001)	0.005 (0.004–0.006; < 0.001)
Waist (cm)	−13.1 (− 17.1–-9.0; < 0.001)	− 11.8 (− 16.7–6.9; < 0.001)	−12.9 (− 20.0 – − 5.9; < 0.001)	−0.003 (− 0.004– − 0.002; < 0.001)	−0.002 (− 0.004– − 0.001; < 0.001)	-0.002 (− 0.004– − 0.000; < 0.012)
Male gender	47.4 (2.1–92.7; < 0.001)	NA	NA	NS	NA	NA

*NA* Not Applicable, *NS* Not Significant

## Discussion

The results obtained for BMC and BMD according to weight status in the different groups classified by BMI z-score showed the highest values in the patients with normal weight and overweight. Both at the lower end of the weight status (underweight) and at the upper end (obese and very obese), both BMC and BMD values decreased. When measuring the percentage of bone mineral mass, a continuous decrease was observed with an increase in weight (except among the underweight and normal weight groups), and this decrease increased with greater obesity. Similarly, logistic regression showed that bone mineralization was inversely related to waist circumference, whereas this relationship was positive for weight and age.

Bone assessment in children and adolescents is not easy as they are in the process of growing, so that height is the most determining factor [11, 12]. Most current research on the possible effect of overweight or obesity on bone mineral supports the presence of a positive association between overweight/obesity and BMD and BMC values [10, 29].. Others find no significant differences between BMC and BMD values after adjusting for weight, lean mass or BMI [32]. Several studies show a positive relationship between the degree of obesity and BMD and BMC [13, 14, 19], which has been called the “obesity paradox” [8, 9]. Kawai et al. interpret that overweight individuals have more muscle and bone mass due to biomechanical reasons (higher body weight), but the increased muscle mass fulfills rather static work leading to lesser strains in bone in relation to individuals with the same muscle mass without overweight. Furthermore, adiposity may influence bone remodeling by secretion of cytokines that influence bone, by production of adipokines that alter the central nervous system, thereby changing sympathetic impulses to bone, and by paracrine effects [33].

Rocher reports that there is an increase in BMD in children with obesity, but it is not sufficient to compensate for the load that the bone must bear due to weight gain [14]. This would support the finding of more frequent fractures [18, 22]. We therefore postulate that the increase found in BMD and BMC in children with overweight could be a regulatory element of the body in response to this increase in weight, although in children with obesity, this compensatory mechanism fails, producing a great decrease in BMD and BMC. In this aspect, it should be noted that the best and simplest marker for clinical practice is waist circumference as a marker of trunk fat, and it is negatively correlated with both BMD and BMC.

Focusing on our main objective, we found that most studies found a positive association between the presence of overweight or obesity and total BMC values (similar to our results), as for example the study by Gallego Suarez et al., which is the largest study and included 8348 children and adolescents of different races [34].

Although no differences were found in our results according to sex, this is under discussion in the literature and no clear results have been found for children or adolescents [35–37]. Nevertheless, these studies only included adolescents and our sample may not have the power needed to detect the differences as we included a wider sample.

One explanation for the few differences found in BMD and BMC values between the normal-weight and overweight/obese groups may be that children and adolescents have a particular subtype of obesity, which has been called metabolically healthy obesity. Individuals with metabolically healthy obesity are those who meet the clinical definition of obesity but have no traditional cardiometabolic risk factors as a consequence of their obesity [38]; nevertheless, the increase in visceral fat measured by DXA has been associated with metabolic risk [39]. This is why our results are especially notable in the most obese subjects.

It should be noted that we had a large sample with 553 subjects, making this one of the largest studies in pediatric populations. In addition, the BMI z-score was included for classifying the children and adolescents according to their weight status, while most studies use total BMI, which is not appropriate in children since the relationship between weight and height changes with age and sex in the first two decades of life. The main limitation of this study is that it was performed in a hospital nutrition and metabolism clinic, so the distribution of the sample included few subjects with underweight and normal weight, in whom the performance of DXA is not included among the studies conducted in routine clinical practice. Our results can therefore be extrapolated mainly to the populations with overweight and obesity.

## Conclusions

Eliminating possible confounding factors, BMC and BMD correlated positively with age and weight and negatively with waist circumference. The bone mineral percentage decreased continuously from the underweight group to the very obese group, implying that the bones of children with obesity must bear a greater relative load the greater the degree of obesity.

## Data Availability

Yes
